# Activation of Haa1 and War1 transcription factors by differential binding of weak acid anions in *Saccharomyces cerevisiae*

**DOI:** 10.1093/nar/gky1188

**Published:** 2018-11-23

**Authors:** Myung Sup Kim, Kyung Hee Cho, Kwang Hyun Park, Jyongsik Jang, Ji-Sook Hahn

**Affiliations:** School of Chemical and Biological Engineering, Seoul National University, Institute of Chemical Processes, 1 Gwanak-ro, Gwanak-gu, Seoul 08826, Republic of Korea

## Abstract

In *Saccharomyces cerevisiae*, Haa1 and War1 transcription factors are involved in cellular adaptation against hydrophilic weak acids and lipophilic weak acids, respectively. However, it is unclear how these transcription factors are differentially activated depending on the identity of the weak acid. Using a field-effect transistor (FET)-type biosensor based on carbon nanofibers, in the present study we demonstrate that Haa1 and War1 directly bind to various weak acid anions with different affinities. Haa1 is most sensitive to acetate, followed by lactate, whereas War1 is most sensitive to benzoate, followed by sorbate, reflecting their differential activation during weak acid stresses. We show that DNA binding by Haa1 is induced in the presence of acetic acid and that the N-terminal Zn-binding domain is essential for this activity. Acetate binds to the N-terminal 150-residue region, and the transcriptional activation domain is located between amino acid residues 230 and 483. Our data suggest that acetate binding converts an inactive Haa1 to the active form, which is capable of DNA binding and transcriptional activation.

## INTRODUCTION

Weak monocarboxylic acids such as acetic, propionic, sorbic and benzoic acid are widely used food and beverage preservatives that prevent microbial cell growth (1–3). However, the inhibitory effect of weak acids on growth is one of the factors that prevent efficient microbial fermentation. Acetic acid and other weak acids present in lignocellulosic hydrolysates prevent the efficient utilization of lignocellulosic biomass (4–6). Furthermore, if the final target product is a weak acid such as lactic acid, high-level production is limited by the product toxicity (7). *Saccharomyces cerevisiae*, which has served as an important model for studies on the responses of yeast to weak acids, is also a promising host for the production of valuable chemicals including lactic acid (8). Therefore, understanding the mechanisms by which yeasts resist weak acids is important for the control of spoilage yeasts that are resistant to weak acid preservatives, and for the generation of robust industrial yeast strains for the production of valuable chemicals.

At low extracellular pH, weak acids mainly exist in the undissociated form, which can readily penetrate cells and dissociate in the neutral cytosol. Acetic acid can also enter cells by facilitated diffusion via aquaglyceroporin Fps1, which is present in the plasma membrane (9,10). The dissociation of acids in the cytosol produces protons and associated counter anions that can cause severe growth inhibition (11). Once the concentration of weak acids builds up, cells must maintain their internal pH and plasma membrane potential within physiological values. Cellular defense mechanisms against weak acid stress include: removing excess cytosolic protons by activating plasma membrane H^+^-ATPase and vacuolar membrane V-ATPase; exporting acid anions through multidrug-resistance (MDR) transporters; and restricting the diffusion of weak acids by remodeling the cell wall and plasma membrane lipid components (12–15). The cytotoxic effects of weak acids and genes induced by them vary depending on their chemical structures. Therefore, cells have regulatory mechanisms that sense structurally different weak acids and induce the appropriate responses. In *S. cerevisiae*, Msn2/Msn4, Rim101, Haa1 and War1 transcription factors are involved in weak acid stress responses (16,17). Msn2 and Msn4 are general stress transcription factors that are activated by multiple environmental stresses (18,19). Rim101 is a transcriptional repressor that is activated by proteolytic cleavage in alkaline environments, which are detected by a plasma membrane pH-sensing complex (17). By contrast, Haa1 and War1 appear to be specific activators of the acid stress response to hydrophilic and lipophilic weak acids, respectively (20–27). However, it is unclear how these transcription factors sense different weak acid stresses.

**Figure 1.**
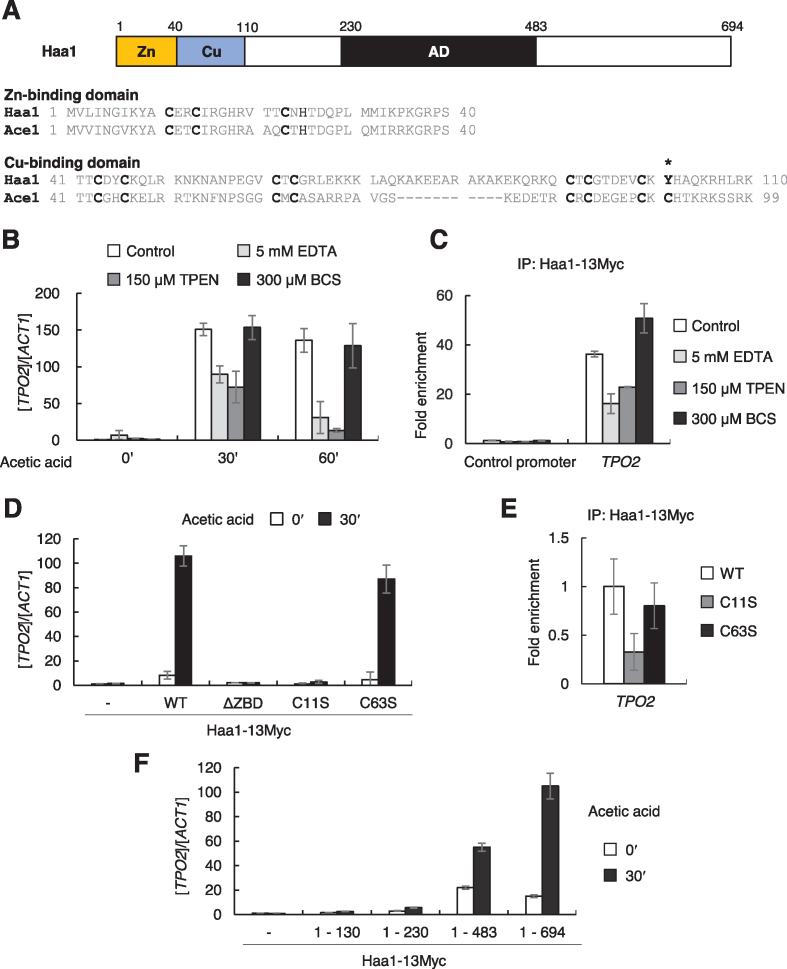
Mapping of the DNA binding and activation domains of Haa1. (**A**) Organization of the predicted Zn-and Cu-binding domains and activation domain in Haa1. Amino acid sequence alignment of Haa1 and Ace1 is shown, where the highly conserved N-terminal Zn-binding domains and moderately conserved Cu-binding domains of Haa1 and Ace1 are compared. (**B**) *S. cerevisiae* BY4741 cells were grown in YPD medium to the exponential phase and pre-treated with chelating agents, 5 mM EDTA, 150 μM TPEN or 300 μM BCS for 2 h, followed by 30 mM acetic acid treatment for the indicated times. mRNA levels of the Haa1 target gene *TPO2* were measured by qRT-PCR and normalized to the mRNA levels of *ACT1*. Each value represents the average ± SD of the relative fold change in expression, normalized to the untreated WT cells (*n* = 3). (**C**) Strain JHY812 expressing Haa1-13Myc was treated with the chelating agents for 2 h, followed by addition of 30 mM acetic acid for 30 min. Binding of Haa1-13Myc to the *TPO2* promoter was detected by ChIP with anti-Myc antibody, and indicated as a fold enrichment relative to the untagged control. Non-transcribed region of Chr. V was used as a control promoter (79). Each value represents the average ± SD from three independent experiments. (**D**) *HAA1* deletion strain harboring the plasmid expressing either wild-type Haa1-13Myc or mutant Haa1-13Myc (ΔZBD (Δ6-40), C11S or C63S) under the control of own promoter was grown in SC-Ura medium to the exponential phase and treated with 30 mM acetic acid for 30 min. mRNA levels of *TPO2* were measured by qRT-PCR and normalized to the mRNA levels of *ACT1*. (**E**) *HAA1* deletion strain expressing Haa1-13Myc wild type or C11S or C63S mutant was treated with 30 mM acetic acid for 30 min, and the DNA binding affinity of Haa1 to the *TPO2* promoter was detected by ChIP with anti-Myc antibody. Fold enrichment of each protein was normalized to that of wild-type Haa1-13Myc. (**F**) Mapping of the activation domain of Haa1. *HAA1* deletion strain harboring the plasmid expressing various C-terminal truncation mutants of Haa1-13Myc under the control of own promoter were grown to the exponential phase and treated with 30 mM acetic acid for 30 min. *TPO2* mRNA levels were measured by qRT-PCR and normalized to the mRNA levels of *ACT1*. Each value represents the average ± SD of the relative fold change in expression, normalized to the untreated control containing empty vector (*n* = 3).

Haa1 was first classified as a fungal Cu-regulated transcription factor based on its protein similarity to the well-known Cu-regulated transcription factor Ace1 (28). The DNA-binding domain of Ace1 consists of a 40-residue Zn-binding domain containing 3 Cys residues and a 70-residue Cu regulatory module containing 8 Cys residues involved in the formation of a polycopper cluster (Figure 1A). Ace1 DNA binding is promoted by the binding of both Cu and Zn ions (29–31), and all 11 Cys residues are critical for either DNA binding or Cu-induced gene expression (32). The Cys residues are also conserved in Haa1 except for the last Cys residue, which is substituted by Tyr in Haa1 (Figure 1A). However, cells grown in Cu-deficient medium do not exhibit any changes in the expression of Haa1 target genes (28), suggesting that Cu-binding is not necessary for Haa1 activity. Instead of Cu-dependent regulation, Haa1 is involved in cellular protection from hydrophilic weak acids such as acetic acid and lactic acid by inducing the transcription of genes such as *TPO2* and *TPO3*, which encode the major facilitator superfamily of transporters involved in the export of acid anions (2,20,22,23). Haa1 rapidly relocates from the cytoplasm to the nucleus during acetic acid- or lactic acid-induced stress, concurrent with some changes to the phosphorylation status (20,33). However, it is not known how Haa1 is activated in response to weak acids.

Lipophilic weak acids such as sorbic acid and benzoic acid disrupt cell membranes, causing effective growth inhibition by passive diffusion (3). Cells confer protection against lipophilic weak acids by activating War1. War1, a transcription factor of the Zn(II)2Cys6 family member, is constitutively localized in the nucleus, even in the absence of stress, and binds to the promoters of its target genes such as *PDR12*, encoding an ABC transporter (27,34–37), but its DNA-binding affinity increases during weak acid stress (38). War1 is hyperphosphorylated during stress, but mutations at the phosphorylation sites have not revealed any essential roles of phosphorylation in War1 activation (25). The lack of evidence of a regulatory War1-activating mechanism suggests that War1 directly binds to acid anions, which is supported by the conformational changes of War1 detected by fluorescence resonance energy transfer (FRET) analysis during treatment with propionic acid (38). However, the direct binding of weak acids to War1 has not yet been confirmed.

In the present study, we used field-effect transistor (FET)-type sensors based on carbon nanofibers (CNFs) (39,40) to confirm that Haa1 and War1 interact directly with various weak acid anions with different affinities. Haa1 preferentially binds to hydrophilic acid anions such as acetate and lactate, whereas War1 preferentially binds to lipophilic acid anions such as sorbate and benzoate. This information reveals the mechanisms by which Haa1 and War1 are differentially activated under various weak acid stresses.

## MATERIALS AND METHODS

### Yeast strains and culture conditions

The yeast strains used in the present study are listed in Table 1. All yeast strains were derived from the BY4741 genetic background. Gene deletion or chromosomal tagging of 13Myc at the C-terminus of Haa1 was performed by using polymerase chain reaction (PCR)-mediated homologous recombination. The yeast cells were grown in YPD medium (1% yeast extract, 2% Bacto Peptone and 2% glucose) or synthetic complete (SC) medium containing 2% glucose, 0.67% yeast nitrogen base without amino acids and 0.2% of an amino acids dropout mixture appropriate for plasmid selection.

**Table 1.** Yeast strains used in this study

Strain	Description	Genotype	Reference
BY4741	WT	*MAT*a *his3Δ1 leu2Δ0 met15Δ0 ura3Δ0*	EUROSCARF
JHY811	*haa1Δ*	BY4741 *haa1Δ:: kanMX6*	This study
JHY812	*HAA1*-*13Myc*	BY4741 *HAA1*-*13Myc::his3MX6*	This study
JHY813	*msn5Δ*	BY4741 *msn5Δ:: kanMX6*	This study
JHY814	*msn5Δ HAA1*-*13Myc*	BY4741 *msn5Δ:: kanMX6 HAA1*-*13Myc::his3MX6*	This study
JHY815	*war1Δ*	BY4741 *war1Δ:: kanMX6*	This study
JHY816	*haa1Δ war1Δ*	BY4741 *haa1Δ:: kanMX6 war1Δ::loxP*	This study

### Plasmids

The plasmids used in the present study are listed in Supplementary Table S1. Plasmids were generated by standard restriction digestion cloning or by site-directed mutagenesis.

### Fluorescence microscopy analysis

Cells transformed with plasmids expressing enhanced green fluorescent protein (eGFP)-tagged Haa1 were grown in SC-Leu medium to the exponential phase, and subjected to 30 mM acetic acid stress for 30 min. Cells were placed in a FluoView FV1000 confocal laser scanning unit with an IX81 inverted microscope (Olympus, Tokyo, Japan), and images were captured with a confocal photomultiplier tube detector. IMARIS software (Bitplane, Belfast, UK) was used to process the confocal images.

### Quantitative reverse transcription PCR (qRT-PCR)

Yeast cells were grown in either YPD or selective SC medium until they had an optical density at 600 nm (OD_600_) of 0.6–0.8, and total RNA was extracted by the hot phenol method. Relative transcription was determined by qRT-PCR, as described previously (41). The primer sequences used for qRT-PCR are listed in Supplementary Table S2.

### Chromatin immunoprecipitation (ChIP)

ChIP was performed using cells expressing Haa1-13Myc, as described previously (41). Cells were inoculated in YPD or selective medium (SC-Ura) to an OD_600_ of 0.2, grown to an OD_600_ of 0.8–1.0, and treated with a final concentration of 1% formaldehyde for 25 min at room temperature, followed by 5 min of quenching with glycine. Cells were collected and washed with cold 150 mM NaCl TBS (50 mM Tris–HCl (pH 7.4), 150 mM NaCl) buffer. Cross-linked yeast cells were re-suspended in lysis buffer (50 mM 4-(2-hydroxyethyl)-1-piperazineethanesulfonic acid (HEPES; pH 7.5), 150 mM NaCl, 1 mM Ethylenediaminetetraacetic acid (EDTA), 1% Triton X-100, 0.1% sodium deoxycholate and protease inhibitors). Sonication was performed in ice-cold water for 10 times for 20 sec each time with 100-s intervals at 24% power output. After 30 min of centrifugation at 4°C, the supernatant was pre-cleared with 20 μl protein A/G beads (Santa Cruz Biotechnology, Dallas, TX, USA) for 2 h, followed by overnight immunoprecipitation with 1 μg of anti-Myc antibody (Santa Cruz Biotechnology, Dallas, TX, USA) and 20 μl protein A/G beads for 1 h to capture the antibodies. Reversal of DNA–protein cross-linking was performed followed by DNA purification using a QIAGEN DNA purification kit (Qiagen, Hilden, Germany). To calculate the fold enrichment of Haa1-13Myc, the bound DNA was analyzed by qPCR using a Roche LightCycle 480 II system and quantified by 2^−ΔΔCT^ method (42). The primer sequences used for qPCR are listed in Supplementary Table S2. The promoter occupancy of Haa1 was normalized by the promoter occupancy of the untagged negative control, and is indicated as fold enrichment.

### Weak acid tolerance test

After dilution the cells with water to OD_600_ values of 1, 0.1, 0.01 and 0.001, 5 μl of the cells was spotted onto a control YPD plate and YPD plates with various concentrations of acetic, lactic, propionic, sorbic or benzoic acid, and incubated at 30°C for 2–3 days.

### Fabrication of carboxyl-functionalized carbon nanofibers (CNFs) and CNF-FET biosensor electrodes

To fabricate carbon nanofibers, electrospun polyacrylonitrile (PAN) fibers were used as the starting materials. First, PAN solution was prepared by dissolving 1.0 g of PAN (MW = 150,000) in 10 ml of *N*,*N*-dimethylformamide (DMF) at 60°C, and the mixture was stirred until the solution became homogeneous. The resulting solution was spun into PAN nanofibers using an electrospinning apparatus (Nano NC, Korea). The diameter of the electrospinning needle was 23 gauge and the voltage was 15 kV, with a flow rate of 10 μl/min. The distance from the needle tip to the grounded collector was fixed at 15 cm. The electrospun PAN nanofibers, were collected and calcined at 270°C for 2 h in air, and carbonized at 800°C for 1 h in flowing nitrogen. Carboxyl-functionalized carbon nanofibers (CNFs) was prepared via oxidation process with strong acid solution. Carbonized PAN nanofibers (250 mg) were soaked in 20 ml of a 3:1 volume mixture of 1 M sulfuric acid (H_2_SO_4_) and 1 M nitric acid (HNO_3_) for 12 h at room temperature with vigorous stirring. The resulting CNF acid mixture was washed with deionized water for several times until the pH of the washing solution became neutral. Finally, the CNFs were collected and dried overnight in a vacuum oven.

To fabricate the CNF-FET electrodes, gold interdigitated array (IDA) electrodes on a glass substrate were prepared by thermal evaporation of Cr/Au (20 nm/200 nm), followed by lift-off process. To build the sensing electrodes, IDA electrodes were treated with 5 wt.% aqueous (3-aminopropyl)triethoxysilane (APTES) solution for 6 h to introduce amine groups to the surface. A mixture of 0.1 wt. % aqueous CNF solution (5 μl) and 1 wt. % aqueous 4-(4,6-dimethoxy-1,3,5-triazin-2-ly)-4-methylmorpholinium chloride (DMTMM, 5 μl) solution were used to treat the aminosilane-modified electrode surfaces for over 6 h at room temperature. The resulting electrodes were carefully rinsed with distilled water to remove excess DMTMM and unbound CNFs. GST, GST-Haa1, GST-Haa1^1–150^, GST-War1 proteins were purified from *Escherichia coli* using glutathione agarose resin and dialyzed in dialysis buffer (50 mM Tris–HCl (pH 7.5), 150 mM KCl, and 15% glycerol). Immobilization of the proteins was carried out in 0.1 M phosphate-buffered saline (PBS, pH 7.4) with 2 μg/l of protein (10 μl) and 1 wt. % aqueous DMTMM solution (10 μl) overnight at 4°C.

### Measurement of real-time responses using CNF-FET electrodes

All the weak acids (acetic, lactic, sorbic and benzoic) were purchased from Sigma-Aldrich (St. Louis. MO, USA). Acid solutions were prepared as 0.1 M stock solutions in PBS (pH 7.4), and titrated to a pH of ∼7 using NaOH solution. Subsequently, the stock solutions were serially diluted to prepare analyte samples, each with a final concentration of 1 fM to 10 μM. Electrical performance of the sensor electrodes was measured by a semiconductor analyzer (Keithley 2612A, Cleveland, OH, USA) and a probe station (MS TECH, model 4000, Seoul, Korea). To monitor the response of CNF-FETs in solution environment, a glass chamber (200-μl volume) was utilized. The chamber was filled with 100 μl of PBS electrolyte (pH 7.4). The gate electrode was immersed in the PBS electrolyte (pH 7.4) and used to bias the sensor to the desired operating point. During the measurement, source-drain bias voltage (*V*_SD_) was maintained at −0.01 V with a gate bias of 0.7 V. The solution (3 μl) containing the analyte was consecutively added into the electrolyte chamber and *I*_SD_ was monitored. The measured *I*_SD_ was normalized as $\Delta I/{\rm{\ }}{I_0} = \ ( {I - {I_0}} )/{I_0}$, where $I$ is the measured real-time current and ${I_0}$ is the initial current.

### Scanning electron microscopy (SEM)

SEM images were obtained using a JSM-6701F microscope (JEOL Ltd.,Tokyo, Japan). For the SEM images of protein-immobilized CNFs, the same procedure was adopted as the CNF-FET electrode fabrication, except that the glass substrate on which the IDA electrode deposited was replaced with a Si wafer.

## RESULTS

### Haa1 requires an N-terminal Zn-binding domain for DNA binding

The N-terminal region of Haa1 is homologous to the DNA-binding domain of Cu-activated transcription factor Ace1, which consists of Zn-binding and Cu-binding domains (Figure 1A). Because Haa1 is not regulated by Cu (28), we investigated the role of the Haa1 Zn-binding domain. First, we determined whether Zn deficiency affects Haa1 target gene transcription during acetic acid stress. When the cells were treated with EDTA, a cell-impermeable metal chelator, to deplete extracellular Zn ions (43), or with *N*,*N*,*N′*,*N′*-tetrakis(2-pyridinylmethyl)-1,2-ethanediamine (TPEN), a cell-permeable Zn chelator (44,45), transcriptional induction of Haa1 target genes *TPO2* and *TDA6* decreased during acetic acid stress compared with the untreated control (Figure 1B and Supplementary Figure S1). However, in agreement with previous studies (28,46), treatment with bathocuproine disulfonic acid (BCS), a cell-impermeable Cu-specific chelator, did not have a significant effect (Figure 1B and Supplementary Figure S1). We also tested the effects of the metal chelators on the DNA binding activity of Haa1 using ChIP assays. In line with the effects of the metal chelators on the transcriptional activation of Haa1 target genes, the chelation of Zn ions, but not Cu ions, significantly reduced the binding of Haa1 to the target promoters during acetic acid stress (Figure 1C and Supplementary Figure S2). We observed comparable Haa1 protein levels in the absence or presence of the chelators, indicating that the effects of Zn depletion are not due to reduced protein expression levels or stability (Supplementary Figure S3).

Because Zn plays an important role in Haa1 activity, we next confirmed the role of the N-terminal 40-residue Zn-binding domain (ZBD) in Haa1 activity. Wild-type Haa1-13Myc and Haa1^ΔZBD^-13Myc lacking 6–40 residues at the N-terminal were expressed in the *haa1*Δ strain to comparable levels (Supplementary Figure S4). However, the *haa1*Δ strain expressing Haa1^ΔZBD^-13Myc exhibited a severe defect in the acetic acid-dependent induction of Haa1 target genes, demonstrating that the predicted N-terminal Zn-binding domain is essential for Haa1 activity (Figure 1D and Supplementary Figure S5).

To further elucidate the role of Zn binding in Haa1 activity, we generated a Haa1^C11S^ mutant, which was able to disrupt zinc–Cys complex formation. Although the protein expression level of Haa1^C11S^ was similar to that of wild-type Haa1 (Supplementary Figure S4), Haa1^C11S^ exhibited severe defects in the transcriptional activation of its target genes (Figure 1D and Supplementary Figure S5), and in binding to the target promoters (Figure 1E and Supplementary Figure S6) during acetic acid stress, indicating that Zn binding to Cys residues including Cys11 is critical for the DNA binding activity of Haa1. In contrast, the mutation of Cys63, a Cys residue corresponding to one of the Cu-binding sites in Ace1, to Ser did not significantly affect the transcriptional activation (Figure 1D and Supplementary Figure S5) or the DNA binding activity of Haa1 (Figure 1E and Supplementary Figure S6), further supporting the Cu-independent regulation of Haa1. Taken together, these results suggest that Zn binding to the N-terminal Zn-binding domain is critical for the DNA binding activity of Haa1.

To determine the minimal Haa1 region sufficient for activation, we progressively deleted the C-terminal portions of the protein (Figure 1F). Although all truncated proteins were expressed to comparable levels (Supplementary Figure S7), Haa1^1–130^ and Haa1^1–230^ did not activate target gene expression, whereas Haa1^1–483^ was active, although to a lesser extent than the full-length Haa1 (Figure 1F). Considering a previous observation that Haa1^1–483^ was able to confer acetic acid tolerance to a similar extent to wild-type Haa1 (47), it seems that the region between amino acids 230 and 483 contains the activation domain required for adaptation to acetic acid stress.

### Haa1 DNA binding is induced during acetic acid stress

**Figure 2.**
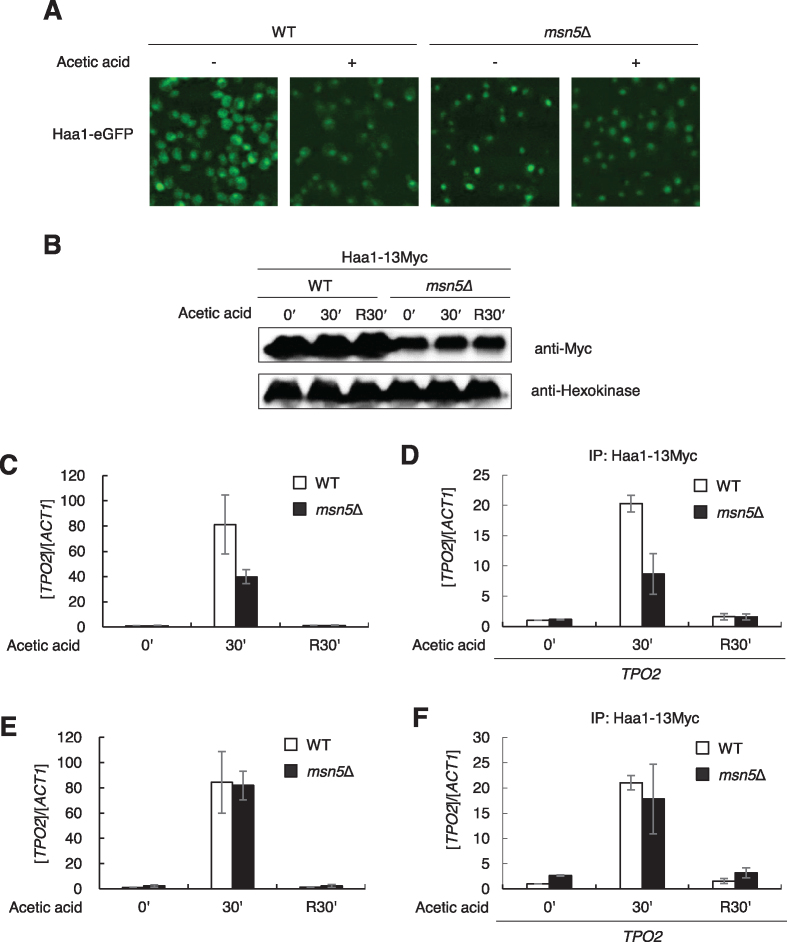
Haa1 DNA binding is induced by acetic acid stress. (**A**) Wild type or *msn5*Δ cells expressing Haa1-eGFP were grown in SC-Leu medium to the exponential phase and 30 mM acetic acid was treated for 30 min. Localization of Haa1-eGFP was analyzed by fluorescence microscope. (**B**) Wild type (JHY812) and *msn5*Δ (JHY814) having Haa1 tagged with 13Myc in the chromosome were grown in YPD medium to the exponential phases and treated with 30 mM acetic acid for 30 min. Cells were then washed and resuspended in fresh YPD medium for another 30 min (R30′). Haa1-13Myc and Hxk1 levels were detected by western blotting with anti-myc antibody and anti-hexokinase antibody, respectively. (**C**) *TPO2* mRNA levels were detected by qRT-PCR. Each value represents the average ± SD of the relative fold change in expression, normalized to the untreated WT cells (*n* = 3). (**D**) Binding of Haa1-13Myc to the *TPO2* promoter was detected by ChIP. Each value represents the average ± SD of the relative fold enrichment, normalized to the untreated WT cells (*n* = 3). (**E**) The qRT-PCR data shown in (C) were normalized with the Haa1 protein levels shown in (B). Protein levels were quantified by using ImageJ program. (**F**) The ChIP data shown in (D) were normalized with the Haa1 protein levels shown in (B).

Haa1 translocates from the cytoplasm to the nucleus during acetic acid or lactic acid treatment (20,33). However, it is unknown whether the nuclear localization of Haa1 is the major regulatory step for its activation. Therefore, we investigated the effect of Haa1 nuclear localization on its activity using an *msn5*Δ mutant, in which Haa1 exists in the nucleus even in the absence of weak acid stress (20). In agreement with the previous study, unlike wild-type cells, where Haa1 translocated to the nucleus during acetic acid stress, the *msn5*Δ cells exhibited nuclear localization of Haa1, even in the absence of stress (Figure 2A). We next determined whether the nuclear localization of Haa1 in *msn5Δ* is sufficient for its activation. We treated wild-type or *msn5*Δ cells expressing Haa1-13Myc with acetic acid for 30 min, then recovered them by resuspending the cells in fresh medium for 30 min. The Haa1-13Myc protein levels in *msn5*Δ were approximately twofold lower than those in the wild-type when the protein levels detected by western blotting were quantified (Figure 2B).

In both the wild-type and *msn5*Δ strains, we only observed a significant induction of *TPO2* expression during acetic acid stress (Figure 2C). In agreement with the transcription data, the ChIP assay revealed that Haa1 bound to DNA during acetic acid stress in an inducible manner in both the wild-type and *msn5Δ* strains (Figure 2D). The transcriptional activation and DNA binding activities of Haa1 during stress exhibited moderately reduced responses in the absence of *MSN5* (Figure 2C and D), which may have been due to the reduced Haa1 protein levels in *msn5*Δ (Figure 2B). After normalizing the qRT-PCR and ChIP data with the different Haa1-13Myc protein levels in the wild-type and *msn5*Δ strains, *msn5*Δ exhibited ∼2–2.6-fold higher Haa1 DNA binding and transcriptional activation of *TPO2* under unstressed conditions, which reflected the low basal DNA binding activity of the nuclear Haa1 in *msn5*Δ (Figure 2E and F). However, even in *msn5Δ*, the basal DNA binding and target gene expression levels were much lower than those induced by acetic acid. Therefore, in the absence of stress, most nuclear Haa1 proteins in *msn5*Δ exist in an inactive form that is unable to bind to DNA. These results demonstrate that the nuclear translocation of Haa1 is not sufficient for its activation, and Haa1 activity is regulated at the level of DNA binding during weak acid stress.

### Activation of Haa1 and War1 by various weak acids

**Figure 3.**
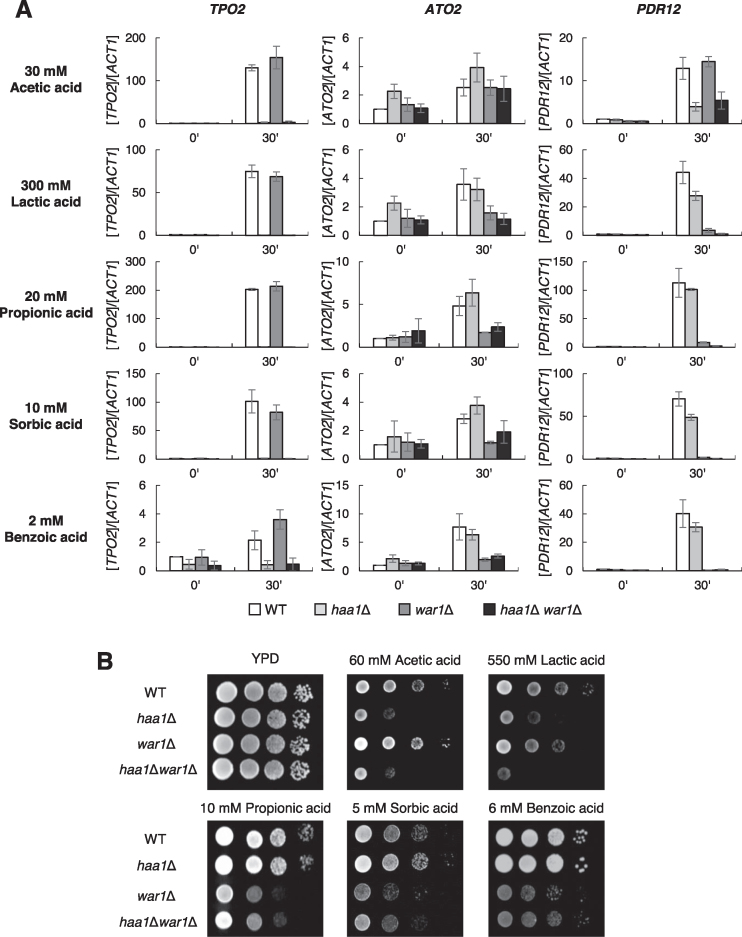
Activation of Haa1 and War1 by various weak acids. (**A**) Wild type, *haa1Δ, war1Δ* and *haa1Δwar1Δ* cells were grown in YPD medium to the exponential phases and treated with 30 mM acetic acid, 300 mM lactic acid, 20 mM propionic acid, 10 mM sorbic acid or 2 mM benzoic acid for 30 min, and mRNA levels of Haa1 and/or War1 target genes, *TPO2, ATO2*, and *PDR12*, were measured by qRT-PCR. Each value represents the average ± SD of the relative fold change in expression, normalized to the untreated wild type cells (*n* = 3). (**B**) Wild type, *haa1Δ, war1Δ* and *haa1Δwar1Δ* cells were grown in YPD medium and then OD_600_ of 1 cells were serially diluted and spotted onto YPD solid medium containing the indicated weak acids.

To understand the activation mechanisms of Haa1, we next investigated how the activation of Haa1 was affected by the identity of the weak acid. The two transcription factors Haa1 and War1 have differential roles depending on the lipophilicity of the weak acids. Previously, it has been shown that Haa1 is activated by hydrophilic weak acids such as acetic acid and lactic acid, whereas War1 is activated by lipophilic weak acids such as sorbic acid and benzoic acid, thereby providing protection against those weak acids (2,20–22,27). However, previous studies have focused on either Haa1 or War1 only, and the experiments were performed under different experimental conditions using different strains with a limited set of weak acids, making it difficult to thoroughly understand the relative contribution of each transcription factor in response to various weak acids. We therefore investigated the activation of both Haa1 and War1 in response to lactic, acetic, propionic, sorbic and benzoic acids. To investigate the activation of each transcription factor under various stress conditions, we monitored the expression of a Haa1-specific target *TPO2, a* War1-specific target *ATO2*, and a shared target *PDR12* in wild-type, *haa1*Δ, *war1*Δ and *haa1*Δ*war1*Δ strains. Owing to the different membrane permeabilities of the weak acids, the higher the lipophilicity of the weak acid, the lower the concentration necessary to induce comparable gene expression levels. Weak acid-dependent induction of *TPO2* was almost completely abolished in *haa1*Δ, but not in *war1*Δ, confirming the Haa1-specific activation of this target gene (Figure 3A). *TPO2* was induced by all the weak acids tested, revealing the activation of Haa1 even by lipophilic weak acids such as sorbic acid and benzoic acid. However, benzoic acid induced the expression of *TPO2* to a much lesser extent than the other weak acids (Figure 3A).

We observed War1-dependent induction of *ATO2* in response to all the weak acids tested except for acetic acid (Figure 3A). These results demonstrate that Haa1 and War1 are commonly activated by a broad range of weak acids, but have different preferences. The activation of *PDR12* exhibited the combined effects of Haa1- and War1-dependent transcription. *PDR12* was induced by acetic acid in a Haa1-dependent manner, in agreement with previous reports (16,17), whereas it was induced by other weak acids in a largely War1-dependent manner with a minor contribution from Haa1 (Figure 3A).

We further investigated the roles of Haa1 and War1 with regard to tolerance of the weak acids tested. In agreement with the previously suggested role of Haa1 in tolerance of hydrophilic weak acids (25,27), *haa1*Δ exhibited reduced tolerance of acetic acid and lactic acid, but not of sorbic acid and benzoic acid (Figure 3B). Therefore, even though Haa1 is activated by sorbic acid and benzoic acid (Figure 3A), Haa1-specific target genes seem to play a minor role in cellular defense against these lipophilic weak acids. In contrast, the role of War1 in weak acid stress tolerance was largely correlated with the transcriptional activation pattern of War1. War1 was involved in tolerance of all the weak acids tested except for acetic acid, which cannot activate War1 (Figure 3B). Although Haa1 is involved in tolerance of propionic acid (2), *war1*Δ exhibited higher susceptibility to propionic acid than *haa1*Δ, suggesting that War1 plays a major role in cellular defense against propionic acid (Figure 3B). A much higher concentration of propionic acid was required to elicit the protective role of Haa1 (Supplementary Figure S8). In the case of lactic acid stress, Haa1 played a major role in stress tolerance, but War1 also contributed to stress tolerance, revealing an increased susceptibility to lactic acid when both genes were deleted (Figure 3B).

In summary, in the *S. cerevisiae* BY4741 strain used in the present study, acetic acid was only able to activate Haa1, but the other weak acids with more than two carbons were able to activate both Haa1 and War1 to different extents. In terms of stress tolerance, Haa1 was required for defense against hydrophilic weak acids (acetic acid and lactic acid), but War1 was required for defense against lipophilic weak acids (propionic acid, sorbic acid, and benzoic acid), and to a lesser extent lactic acid. Our results largely agree with the previously suggested roles of Haa1 and War1 in weak acid stress responses, but provide more comprehensive evidence regarding the types of weak acids involved in their activation, and their roles in defense against each weak acid.

### Direct binding of acetate to Haa1 detected using the CNF-FET biosensor

**Figure 4.**
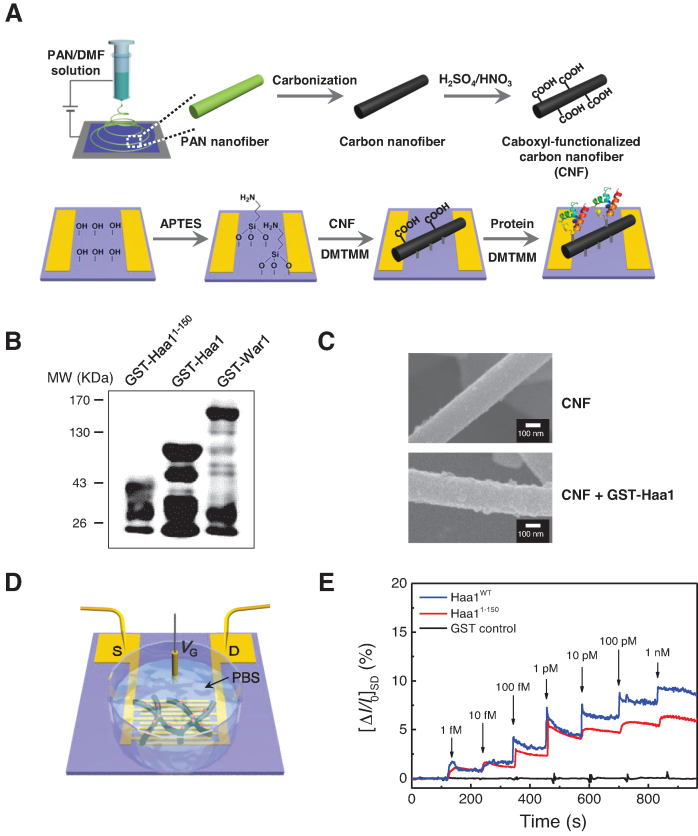
Detection of the direct binding of acetate to Haa1 using the CNF-FET biosensor. (**A**) Schematic diagram of the fabrication process of the CNF-FET biosensor electrodes. (**B**) GST-tagged proteins used to fabricate the CNF-FET biosensor. Proteins were purified from *E. coli* and detected by western blotting with anti-GST antibody. **(C)** Scanning electron microscopy (SEM) images of the CNF surfaces before and after the immobilization of GST-Haa1 proteins. (**D**) Schematic illustration of the CNF-FET biosensor with ionic liquid gate for solution measurements. (**E**) Receptor (GST-Haa1^WT^, GST-Haa1^1–150^ and GST control)-dependent sensing responses of the CNF-FET devices to various concentrations of acetic acids (1 fM to 1 nM).

The activation of Haa1 and War1 by a wide range of weak acids suggests that these transcription factors sense different weak acids by direct binding. To detect protein–weak acid interactions, we used a FET-type sensor based on carbon nanofibers (CNF-FET), which enables label-free, real-time monitoring of interactions with high sensitivity (48,49). Figure 4A shows the fabrication process of the CNF-FET biosensor electrodes. The electrodes comprise a gold microelectrode consisting of several pairs of source (S) and drain (D) electrodes, and transducer materials (CNF), where the proteins are conjugated. The electrode surfaces were modified with aminosilane to ensure a good electrical contact between the electrode substrate and the transducer materials deposited onto them (50). The CNFs were oxidized with strong acids to form carboxylic groups on their surfaces. The condensation reaction between the amine groups on the electrode substrates and the carboxylic acids on the CNFs resulted in covalent immobilization of the CNFs on the substrate. We also used this covalent bonding strategy to attach GST-tagged Haa1 proteins purified from *E. coli* (Figure 4B) to the CNF transducer. We confirmed the immobilization of GST-Haa1 on the CNFs by SEM (Figure 4C). The protein-modified CNFs had relatively rough surfaces compared with the smooth pristine CNFs.

To characterize the electrical properties of the CNF-FET electrodes, we constructed a ionic liquid-gated FET configuration in PBS (pH 7.4), which was used as the electrolyte that can provide solution gate control. Figure 4D illustrates the set-up of the fabricated device and sensor. A detailed experimental set-up is given in Supplementary Figure S9. In this transistor device-coupled biosensor, the current flow between the S and D electrodes (*I*_SD_) via CNF can be changed when the protein–analyte interaction changes the charge distribution on the surface of the CNFs, which acts like an indirect gate potential. Therefore, this biosensor directly converts a biological signal into an electrical signal through the changes in the conductivity of the CNFs. The characterization of the Haa1-functionalized CNF-FET electrodes is described in the supplementary data (Supplementary Figure S10). Both the output and transfer curves indicate that the device exhibits n-type semiconducting characteristics, in which the charge carriers are the electrons that have accumulated on the surface of the CNF when a positive gate voltage is applied through the solution gate. The interaction between the proteins (Haa1) and the particular target molecules can change the charge carrier density, resulting in current (*I*_SD_) changes.

We carried out real-time sensing measurements using the CNF-FET biosensors to determine whether Haa1 is regulated by direct interaction with the weak acids. As shown in Figure 4E, the real-time response of the FET-type device was examined by monitoring current (*I*_SD_) changes during exposure to various concentrations of acetic acid solution adjusted to pH 7.0. Because the p*Ka* of acetic acid is 4.76, dissociated acetate anions are dominant in the experimental solution at pH 7.4. The biosensor functionalized with full-length GST-Haa1 protein exhibited immediate current increase after adding a wide range of concentrations of acetate solution ranging from 1 fM to 1 nM (Figure 4E). Owing to the high aspect ratio and surface area of the CNFs, high amount of receptors are loaded per unit area, which allows detection of more target molecules at a time with high sensitivity (51). Additionally, the nanoscale dimensions of CNFs lead to rapid depletion or accumulation of charge carriers within the nanowires, allowing rapid detection of subtle changes in the surroundings (52,53). Therefore, the response time was very short (3 s), and we were able to detect a remarkably low concentration (1 fM) of acetate. However, the control electrode immobilized with GST protein did not exhibit any perceptible changes after the introduction of acetate, confirming that the current changes were generated by the specific interaction between Haa1 and acetate (Figure 4E). The direct binding of acetate to Haa1 suggests that Haa1 can be activated by directly sensing weak acid anions. The mechanism underlying the current increase following the binding of the acetate to Haa1 can be explained by a charge carrier accumulation theory (54,55) (Supplementary Figure S11).

We also attached truncated GST-Haa1^1–150^ proteins to the CNF-FET electrode to investigate the acetate-binding site (Figure 4B). The response of Haa1^1–150^ to acetate was similar to the response exhibited by the wild-type, but with slightly smaller current changes (Figure 4E). These results demonstrate the direct binding of the acetate in the 1–150 amino acid region of the N-terminal of Haa1.

### Differential binding of weak acid anions to Haa1 and War1

**Figure 5.**
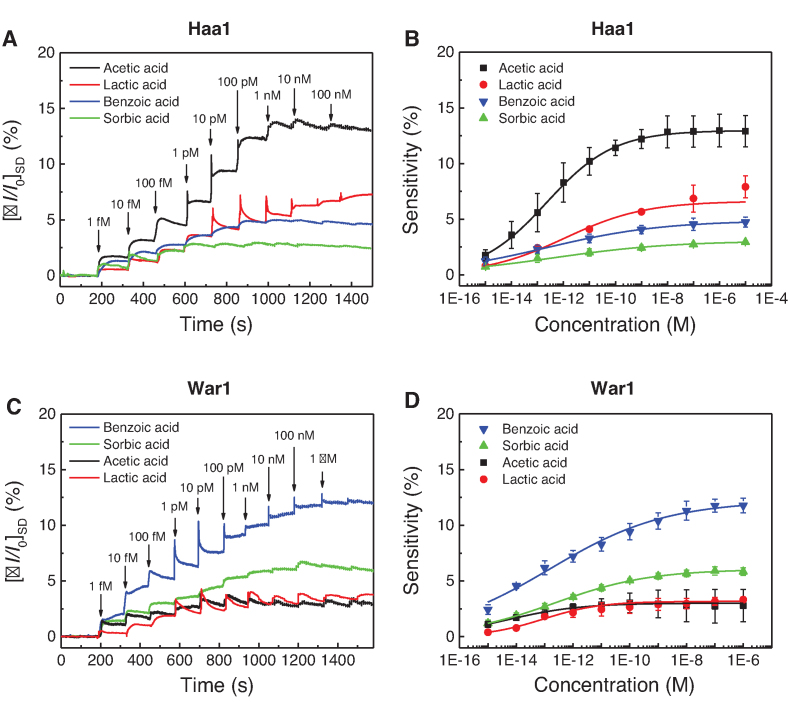
Direct binding of various weak acids to Haa1 and War1. (**A**) Real-time response of the Haa1-functionalized CNF-FET biosensor to various concentrations of acetic, lactic, sorbic and benzoic acids (1 fM to 100 nM). (**B**) Concentration-dependent responses to acetic, lactic, sorbic and benzoic acids with normalized sensitivity. Normalized sensitivity indicates the saturation level of ${[ {I/{I_0}} ]_{SD}} \times \ 100$ measured after exposure to the acid analytes. The real-time response measurement was repeated three times and the sensitivity is indicated as average ± SD. (**C**) Real-time response of the War1-functionalized CNF-FET biosensor to various concentrations of acetic, lactic, sorbic and benzoic acids (1 fM to 1 μM). (**D**) Concentration-dependent responses to acetic, lactic, sorbic and benzoic acids with normalized sensitivity. The real-time response measurement was repeated three times.

To investigate the differences in the reactivity of Haa1 toward various weak acids, we determined the real-time responses of three other weak acids besides acetic acid. We detected concentration-dependent current changes when lactate, sorbate, and benzoate solutions were injected into the Haa1-functionalized CNF-FET sensor electrodes, suggesting the direct binding of these anions to Haa1 (Figure 5A and B). The sensitivity of the CNF-FET was greatest in response to acetate, followed by lactate, benzoate, and sorbate. However, sensitivity to lactate, sorbate, or benzoate was much lower than sensitivity to acetate (Figure 5A and B). These results agree with the more prominent role of Haa1 in response to acetic acid and lactic acid than to the more lipophilic weak acids.

Using the CNF-FET sensor functionalized with GST-War1 protein (Figure 4B), we next determined whether War1 can also directly bind to weak acid anions. As with the Haa1-functionalized CNF-FET sensor, we detected concentration-dependent changes in *I_SD_* when we investigated weak acid anions using the War1-functionalized CNF-FET sensor, suggesting their direct binding to War1 (Figure 5C and D). In contrast to Haa1, War1 was most sensitive to benzoate followed by sorbate. War1 had much lower sensitivity to acetate and lactate, reflecting the preference of War1 for the more lipophilic weak acid anions. Although we did not detect the transcriptional activation of War1 during acetic acid stress *in vivo* (Figure 3A), the War1-functionalized CNF-FET sensor exhibited weak *I_SD_* changes in response to acetate (Figure 5C and D). Considering the high deviations in sensitivity to acetate in three repeated experiments (Figure 5D), acetate binding to War1 was less significant compared with the binding of the other weak acids. However, it is also possible that acetate binds to War1 with low affinity, but cannot induce sufficient conformational changes to activate War1. Both Haa1 and War1 are activated by the direct binding of weak acid anions, but the sensitivity depends on the structure of the weak acid anions, which is responsible for the differential activation of Haa1 and War1 and induction of proper cellular responses to the weak acids.

## DISCUSSION

### Activation of Haa1 and War1 by direct and differential binding of weak acid anions

Cellular adaptation to weak organic acids is fundamental to cell survival and proliferation, but much remains unanswered regarding how cells sense such insults. In *S. cerevisiae*, Haa1 and War1 transcription factors are involved in cellular protection from hydrophilic and lipophilic weak acids, respectively (56). Although their target genes and activating weak acid signals have been studied extensively, it has long been questioned how Haa1 and War1 are differentially activated depending on the identity of the weak acid. In the present work, using a CNF-FET biosensor, which allows sensitive real-time detection of protein–small molecule interactions, we obtained evidence that Haa1 and War1 are activated by the direct binding of weak acid anions with different binding affinities.

CNF-FET is an effective technique for studying the electrochemical bioaffinity between receptors and target molecules (52). As a transducer material, CNF is a 1D nanostructure that enables anisotropic electronic properties and simple integration with two terminal microcircuits (57,58). Even small amounts of material are sufficient to obtain adequate responses. Because the CNF-FET biosensor device is a label-free sensing system, it is easy to fabricate and produces good electric signals without altering protein activity. In particular, the ionic liquid-gated type FET, which allows sensing experiments to be conducted in the liquid phase, can be used to observe microscopic changes during receptor and analyte interaction in real time. Furthermore, because small current changes can be amplified owing to the induced electric field, it is possible to perform sensitive analyses to detect very low concentrations of analytes (59,60).

We showed that both Haa1 and War1 can directly bind to various weak acid anions. The concentration-dependent sensitivity allowed us to detect affinity differences among the weak acids. The Haa1 and War1 proteins were most sensitive to acetate and benzoate, respectively (Figure 5), which is consistent with their roles in cellular protection against hydrophilic and lipophilic weak acids, respectively (Figure 3). Furthermore, in combination with our results showing the activation of Haa1 and War1 by a wide range of weak acids (Figure 3A), Haa1 and War1 bind to different weak acid anions with different affinities, thereby triggering the appropriate gene expression required for defense against each weak acid. However, although our results showed that sorbate and benzoate can bind to Haa1 (Figure 5A and B) and activate its transcriptional activity (Figure 3A), Haa1 activation seems to be dispensable for cellular protection against these weak acids (Figure 3B). Haa1 is directly or indirectly involved in activation of approximately 80% of the 112 acetic acid-inducible genes (23). However, so far, only *PDR12* is known to be regulated by both Haa1 and War1 (16). The very limited overlap between the Haa1- and War1-regulated genes suggests that Haa1 and War1 have specific roles against different weak acid stress conditions. Therefore, *S. cerevisiae* Haa1 might be mainly responsible for sensing acetate stress, as previously appreciated (2,61), but seemingly unnecessary activation by sorbate and benzoate might be due to the low binding specificity of weak acid anions to Haa1. However, in other yeast species, where Haa1 and War1 regulate a different set of genes, activation of Haa1 by lipophilic weak acids could be part of the defense mechanisms against these weak acids. In fact, a Haa1 homologue in *Zygosaccharomyces bailii* (*Zb*Haa1) is involved in the tolerance of sorbic and benzoic acid as well as acetic acid (62,63). The sensitivity of the Haa1-functionalized CNF-FET to acid anions decreased in the order: acetate, lactate, benzoate and sorbate (Figure 5A and B), which does not correlate with the order of hydrophilicity of these acid anions. The hydrophobic constant (log *P*), which reflects lipophilicity, increases in the order: lactic acid (−0.72), acetic acid (−0.17), sorbic acid (1.33) and benzoic acid (1.87) (64). Therefore, lipophilicity is not the major factor determining the binding specificity of weak acid anions to Haa1.

In contrast, War1 is activated by weak acids with more than two carbons (lactic, propionic, sorbic, and benzoic acids), and contributes to tolerance of these weak acids (Figure 3). In agreement with its protective role against more lipophilic weak acids (27,38), the War1-functionalized CNF-FET was most sensitive to benzoate followed by sorbate (Figure 5C and D). War1 belongs to the Zn(II)2Cys6 family of transcription factors, which includes several transcription factors that are activated by the direct binding of their ligands. For example, Pdr1 and Pdr3, which are involved in multi-drug responses, are activated by direct interaction with structurally diverse xenobiotics (65), and Put3, which is involved in proline utilization, is activated by direct interaction with proline to adapt to nitrogen starvation (66). Leu3, which is involved in branched-chain amino acid synthesis (67), is regulated by the direct binding of alpha-isopropylmalate (IPM), which accumulates as the first product in leucine biosynthesis during leucine starvation (68–70). The direct binding of IPM to the middle region of Leu3 stimulates the activation of Leu3 by interfering in the intramolecular interaction between the middle region and the activation domain of Leu3 (70,71). Therefore, our findings further support the commonly observed regulatory mechanisms of many Zn(II)2Cys6 family transcription factors that act by directly binding to small-molecule ligands. Sensor experiments can detect direct interactions between proteins and ligands, but cannot reveal the molecular details of these interactions. Future investigations will be required to clarify the exact binding sites in Haa1 and War1, and the precise mechanisms by which proteins and weak acid anions bind.

### Mechanisms by which weak acids regulate Haa1

Haa1 is a paralogue of Ace1 and has almost the same pattern of Cys residues in the N-terminal Zn-binding and Cu-binding domains (28). However, they have evolved to play distinct roles by sensing different ligands for activation. Whereas Ace1 is activated by the binding of Cu(I) to the N-terminal Cys residues, our study revealed that Haa1 is activated by the direct binding of weak acid anions, especially acetate. Ancestral Haa1 homologues found in yeast species of pre-whole genome duplication (WGD) events, such as *Z. bailii*, can be activated by both Cu and acetic acid (72). In *S. cerevisiae*, dual-functioning *Zb*Haa1 is evolutionarily separated into two different transcription factors, Haa1 and Ace1, retaining the N-terminal Zn-binding domain required for DNA binding. The Zn-binding domain is also conserved in another Cu-regulated transcription factor, Mac1 (73,74). We demonstrated that the Zn-binding domain is essential for Haa1 DNA binding activity (Figure 1). However, considering the different DNA binding motifs of Haa1 (5′-(G/C)(A/C)GG(G/C)G-3′) and Ace1 (5′-TC(T)4–6GCTG-3′), further C-terminal regions might be involved in determining the DNA binding specificity of Haa1 (23,75).

Ace1 exists in the nucleus independently of the Cu status, and Cu(I) binding converts an inactive Ace1 to a functional protein capable of DNA binding and activating transcription (30,76,77). In contrast, Haa1 translocates from the cytoplasm to the nucleus by an as yet unidentified mechanism during weak acid stress (20,33). Haa1, which was localized in the nucleus in the *msn5*Δ mutant, exhibited reduced stability (Figure 2B). This has also been reported for another transcription factor Msn2, which also translocates from the cytosol to the nucleus during stress, and accumulates in the nucleus in the *msn5*Δ mutant (78). The destabilization of nuclear Msn2 has been suggested as one of the regulatory mechanisms of Msn2 activity (78). We showed that nuclear localization is not sufficient for Haa1 to bind DNA without acetic acid stress (Figure 2). Therefore, the conformational changes to Haa1 induced by acetate binding might convert the inactive Haa1 to an active form that is capable of binding to DNA, thereby activating the transcription of its target genes. We mapped the activation domain between amino acid residues 230 and 483 in Haa1 (Figure 1F). Furthermore, the acetate-binding site is located within amino acid residues 1–150 based on our CNF-FET biosensor experiment (Figure 4E). More detailed identification of the acetate-binding sites and the structural changes induced by acetate-binding is necessary to further understand the activation mechanisms of Haa1.

## Supplementary Material

Supplementary Data
