# Supplementary material for: Activation of Haa1 and War1 transcription factors by differential binding of weak acid anions in Saccharomyces cerevisiae
Source: Nucleic Acids Res. 2018 Nov 23;47(3):1211–24. doi: 10.1093/nar/gky1188 (PMC6379682; doi:10.1093/nar/gky1188)
Supplement: Supplementary Data [file gky1188_supplemental_files.pdf]

**Supplementary data**

# Activation of Haa1 and War1 transcription factors by differential binding of weak acid anions in *Saccharomyces cerevisiae*

Myung Sup Kim, Kyung Hee Cho, Kwang Hyun Park, Jyongsik Jang*, and Ji-Sook Hahn*

School of Chemical and Biological Engineering, Seoul National University, Institute of Chemical Processes, 1 Gwanak-ro, Gwanak-gu, Seoul 08826, Republic of Korea

The authors wish it to be known that, in their opinion, the first 2 authors should be regarded as joint First Authors.

*To whom correspondence should be addressed

Phone: +82-2-880-9228

Fax: +82-2-888-1604

E-mail: hahnjs@snu.ac.kr

Correspondence may also be addressed to

Phone: +82-2-880-7069

Fax: +82-2-888-1604

E-mail: jsjang@snu.ac.kr

# Supplementary Tables

## **Table S1.** Plasmids used in this study

| Plasmid | Description | Reference |
|---|---|---|
| **Plasmids for *E. coli*** | | |
| pGEX4T-1-HAA1 | *HAA1* cloned between SmaI and XhoI of pGEX4T-1 | This study |
| pGEX4T-1-WAR1 | *WAR1* cloned between SmaI and XhoI of pGEX4T-1 | This study |
| **Plasmids for *S. cerevisiae*** | | |
| p416TEF | CEN/ARS plasmid, $P_{TEF1}$, $T_{CYC1}$, URA3 marker | (1) |
| pUG27 | Plasmid containing loxP-HIS5-loxP deletion cassette | EUROSCARF |
| pSH47 | Plasmid containing a gene for Cre-recombinase, URA3 marker | EUROSCARF |
| p416-$P_{HAA1}$-HAA1-13Myc-$T_{ADH1}$ | *HAA1* ORF with own promoter (600 bps) and 13 Myc tag with ADH1 terminator (250 bps) cloned between Not1 and Xho1 of pRS416 | This study |
| p416-$P_{HAA1}$-HAA1$^{DBD\Delta}$-13Myc-$T_{ADH1}$ | Deletion of *HAA1*$^{6\text{-}40}$ from p416-$P_{HAA1}$-HAA1-13Myc- $T_{ADH1}$ | This study |
| p416-$P_{HAA1}$-HAA1$^{1\text{-}130}$-13Myc-$T_{ADH1}$ | Deletion of *HAA1*$^{131\text{-}694}$ from p416- $P_{HAA1}$-HAA1-13Myc-$T_{ADH1}$ | This study |
| p416-$P_{HAA1}$-HAA1$^{1\text{-}230}$-13Myc-$T_{ADH1}$ | Deletion of *HAA1*$^{231\text{-}694}$ fromp416- $P_{HAA1}$-HAA1-13Myc-$T_{ADH1}$ | This study |
| p416-$P_{HAA1}$-HAA1$^{1\text{-}483}$-13Myc-$T_{ADH1}$ | Deletion of *HAA1*$^{484\text{-}694}$ from p416- $P_{HAA1}$-HAA1-13Myc-$T_{ADH1}$ | This study |
| p416- $P_{HAA11}$-HAA1$^{C11S}$-13Myc-$T_{ADH1}$ | *HAA1* $^{C11S}$ mutation from p416- $P_{HAA1}$-HAA1-13Myc-$T_{ADH1}$ | This study |
| p416- $P_{HAA1}$-HAA1$^{C63S}$-13Myc-$T_{ADH1}$ | *HAA1* $^{C163S}$ mutation from p416- $P_{HAA11}$-HAA1-13Myc-$T_{ADH1}$ | This study |
| p415ADH-HAA1-eGFP | *HAA1* ORF cloned between Spe1 and Xho1 of pRS415 ADH | This study |

**Table S2.** List of PCR primers

| Primer names | Sequence (5'-3') |
|---|---|
| **qRT-PCR** | |
| *TPO2* | CATATTATTTGGGTTGGTCCAGCTTC |
| | GCTGCCTTCCCCTTGTTTACCATTC |
| *TDA6* | GTATATCGGAGGTCCTCGTGGCC |
| | GTGATAATTGTGGTGTCCTACTCTATAC |
| *ATO2* | CTATCCATTGCAAACTTCACAGGCG |
| | CATTATGGTCCATCCATTCGCATTACC |
| *PDR12* | CAATATTGCCGCTATGTTGATTTGTTAC |
| | CATTTTCCAAACAGTTCCAGGTGACG |
| **ChIP** | |
| *TPO2* | CCGAGTGAAATATACTGCTTAACTGTGTCC |
| | CAATGGGCGCGCTTACTCAAATTCCC |
| *TDA6* | GTCCGATTGGGCTCCAGTAAACC |
| | CTGGTCTTTAGCACACGACGACCGTAC |
| Control promoter | CCGATTTGTGAGATTCTTCCTGGCTG |
| (Chr. V) | GTGATGGTGATAGGCGTTGAGTATGTG |

## Supplementary Figures

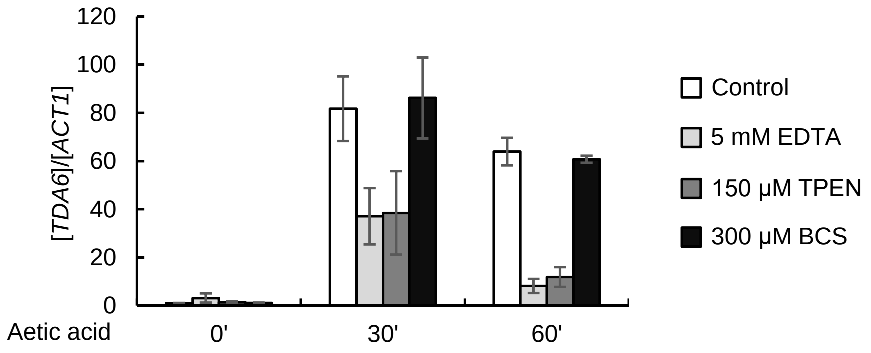


**Figure S1. Transcription levels of Haa1 target gene *TDA6* upon the treatment of metal chelators.** *S. cerevisiae* BY4741 cells were grown to the exponential phase in YPD medium and pre-treated with chelating agents, 5 mM EDTA, 150 μM TPEN, or 300 μM BCS for 2 h, followed by 30 mM acetic acid treatment for the indicated times. mRNA levels of Haa1 target genes *TDA6* were measured by qRT-PCR and normalized to the mRNA levels of *ACT1.*

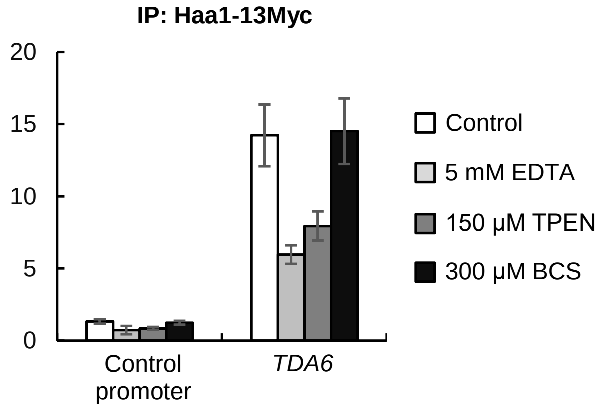


**Figure S2. Binding of Haa1-13Myc to the *TDA6* promoter upon the treatment of metal chelators.** Strain JHY812 expressing Haa1-13Myc was treated with the chelating agents for 2 h, followed by addition of 30 mM acetic acid for 30 min. Binding of Haa1-13Myc to the *TDA6* promoter was detected by ChIP with anti-Myc antibody, and indicated as fold enrichment relative to the untagged control. Non-transcribed region of Chr. V was used as a control promoter (2).

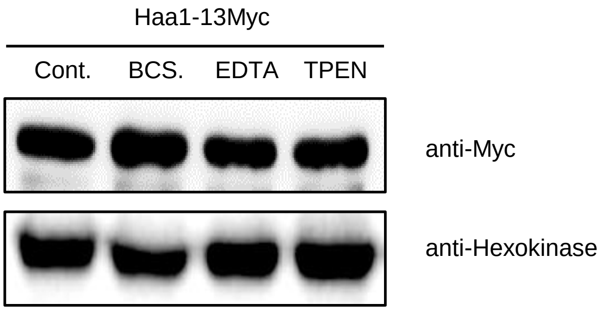


**Figure S3. Haa1 protein levels upon the treatment of metal chelators.** Strain JHY812 expressing Haa1-13Myc was treated with chelating agents, 5 mM EDTA, 150 μM TPEN, or 300 μM BCS, for 2 h followed by 30 mM acetic acid for 30 min. Western blotting was performed as previously described (3,4) by using anti-Myc antibody (Santa Cruz Biotechnology) and anti-Hexokinase antibody (US Biological). To detect protein levels of Haa1s, cells were grown in YPD medium to the exponential phase and lysed in IP150 buffer [50 mM Tris-HCl (pH 7.4), 150 mM NaCl, 2 mM $MgCl_2$, 0.1% NP-40 containing 0.1% protease inhibitor cocktail (Calbiochem) and 1 mM phenylmethylsulfony fluoride (Calbiochem)] followed by incubation with suitable antibodies to detect protein levels. Hexokinase was used as a loading control.

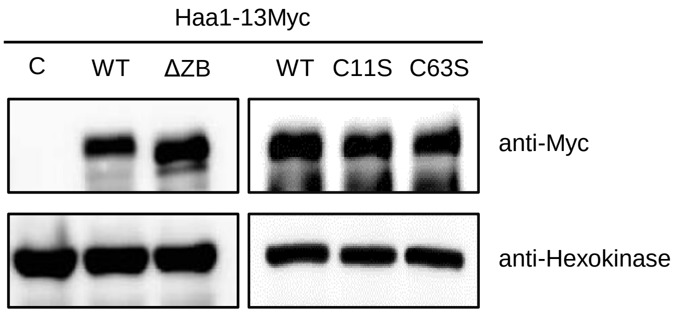


**Figure S4. Protein levels of Haa1$^{\text{WT or ΔZBD or C11S or C63S}}$.** *HAA1* deletion strain JHY811, expressing either wild-type Haa1-13Myc or Haa1$^{\text{ΔZBD (6-40Δ), C11S, or C63S}}$-13Myc under the own promoter were grown to exponential phases. Cells were lysed in IP150 buffer and 100 μg of protein was used to detect protein levels by western blotting.

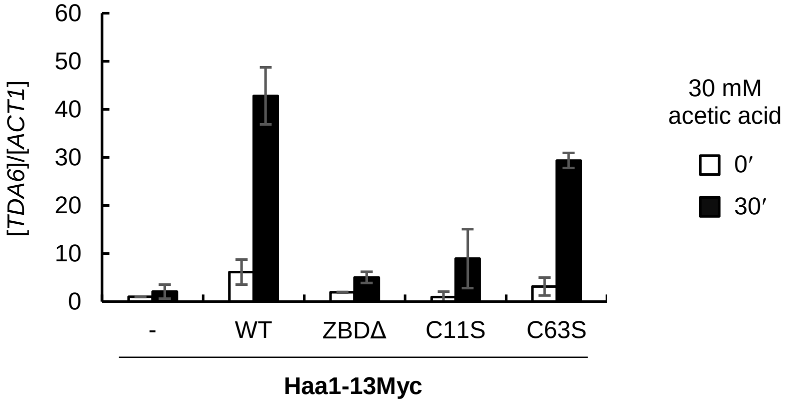


**Figure S5. Roles of the Zn-binding domain of Haa1 in transcriptional activation of the Haa1 target gene *TDA6*.** *HAA1* deletion strain harboring plasmid expressing either wild-type Haa1-13Myc or mutant Haa1-13Myc (ΔZBD (Δ6-40), C11S, or C63S) under the control of own promoter was grown in SC-Ura medium to the exponential phase and treated with 30 mM acetic acid for 30 min. mRNA levels of Haa1 target genes *TDA6* were measured by qRT-PCR and normalized to the mRNA levels of ACT1.

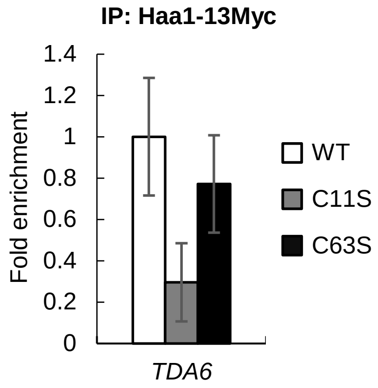


**Figure S6. Binding of Haa1 $^{\text{WT or C11S or C63S}}$ -13Myc to the *TDA6* promoter.** *HAA1* deletion strain expressing Haa1-13Myc wild type or C11S or C63S mutant was treated with 30 mM acetic acid for 30 min, and the DNA binding affinity of Haa1 to the *TDA6* promoter was detected by ChIP with anti-Myc antibody. Fold enrichment of each protein was normalized to that of wild-type Haa1-13Myc.

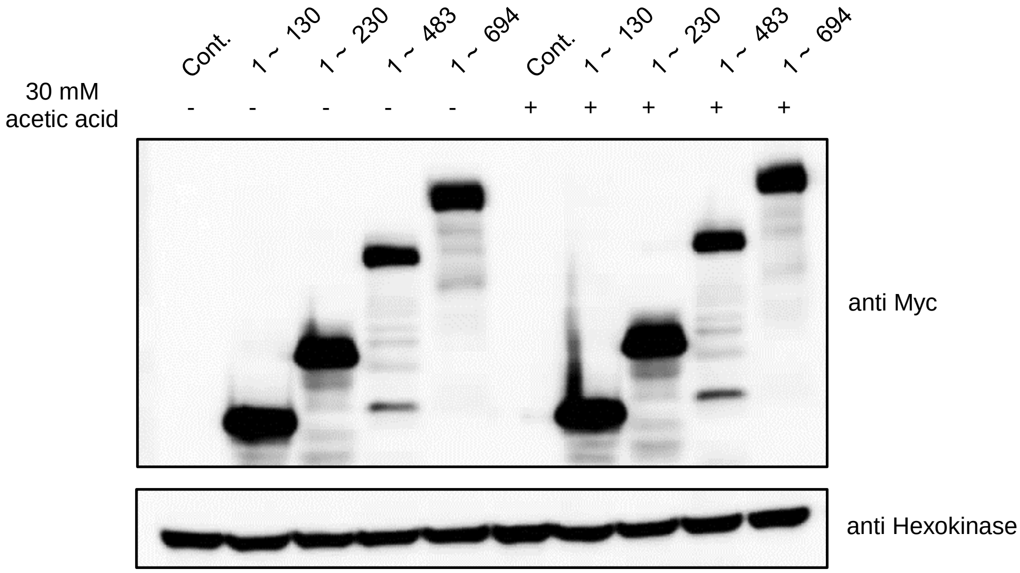


**Figure S7. Protein levels of various truncated Haa1.** *HAA1* deletion strain JHY811 harboring plasmid expressing Haa1$^{1\text{-}130,\ \text{or}\ 1\text{-}230,\ \text{or}\ 1\text{-}483,\ \text{or}\ 1\text{-}694}$-13Myc from the own promoter were grown to the exponential phase and sampled with or without the treatment of 30 mM acetic acid for 30 minutes. Cells were lysed in IP150 buffer and 100 μg of protein was used to detect protein levels by western blotting.

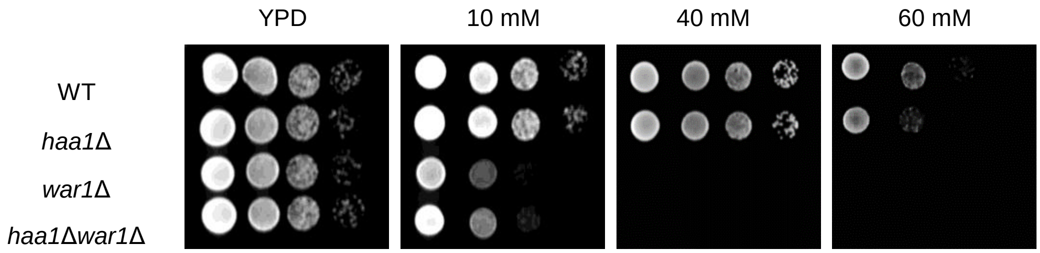


**Figure S8. Growth inhibition by various concentrations of propionic acid.** Wild type, *haa1Δ*, *war1Δ*, and *haa1Δwar1Δ* cells were grown in YPD medium and then $OD_{600}$ of 1 cells were serially diluted and spotted onto YPD solid medium with or without 10, 40, or 60 mM propionic acid.

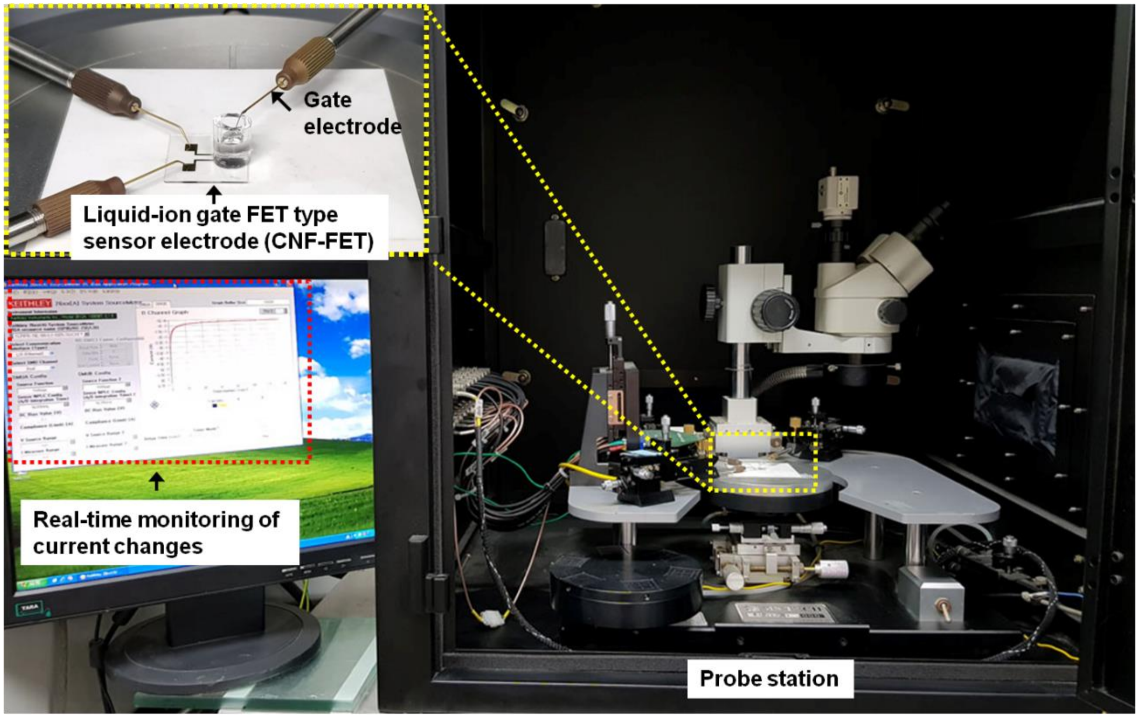


**Figure S9. The overall setup of the sensor system to measure the I-V curves in real-time with the probe station and the computer.** The current changes are measured by the source meter connected to the probe station. Once weak acids are applied into the liquid electrolyte of the sensor electrode, changes in the electrical currents are displayed.

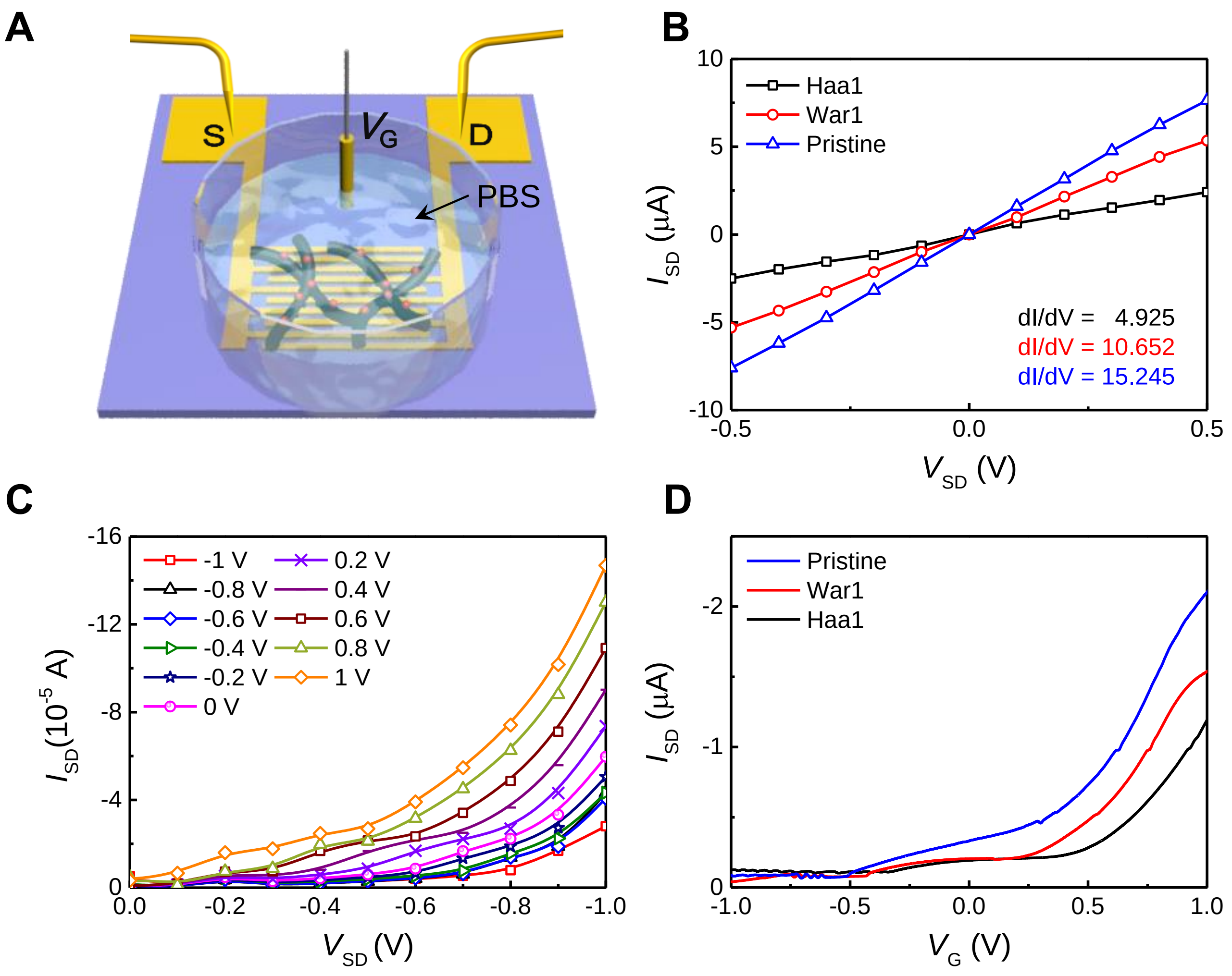


**Figure S10. Electrical properties of CNF-FET biosensor electrodes. (A)**. Schematic illustration of the CNF-FET biosensor with liquid ion gate for solution measurements. **(B)** Current-voltage (I-V) curves of the CNFs on the electrode substrate before and after protein immobilization over a range of -0.5 to +0.5 V. $I_{SD}$-$V_{SD}$ graphs show the characteristics of contacts between the semiconductor and the electrode. Whether the graph is linear or not, it can be used to determine the stability of contacts. The d*I*/d*V* value slightly decreased after anchoring proteins on CNF, which might be due to the accumulation of negative charges (electrons) on the CNF surfaces owing to the proteins. However, the *I-V* graphs maintained linear slope, indicating that stable ohmic contact was preserved during the protein attachment. It can be assumed that the

contact resistance is negligible and signal change occurs only by electrostatic gating. **(C)** $I_{SD}$-$V_{SD}$ output curves of the CNF-FET biosensor (VG from -1.0 to +1.0 V in a step of 0.1 V at $V_{SD}$ scan rate of -100 mV). The source-drain current ($I_{SD}$) negatively increase as $V_G$ positively increases. **(D)** Transfer curves of the CNF-FETs with or without proteins. The transistor characteristics of the protein-immobilized CNT-FET device was evaluated by $I_{SD}$-$V_G$ transfer curve. Slight variation in dI/dV values and transfer curves (B and D, respectively) infer that covalent amide bonding between the protein and CNFs has a certain effect on the electrical properties of CNF-FET biosensor electrodes.

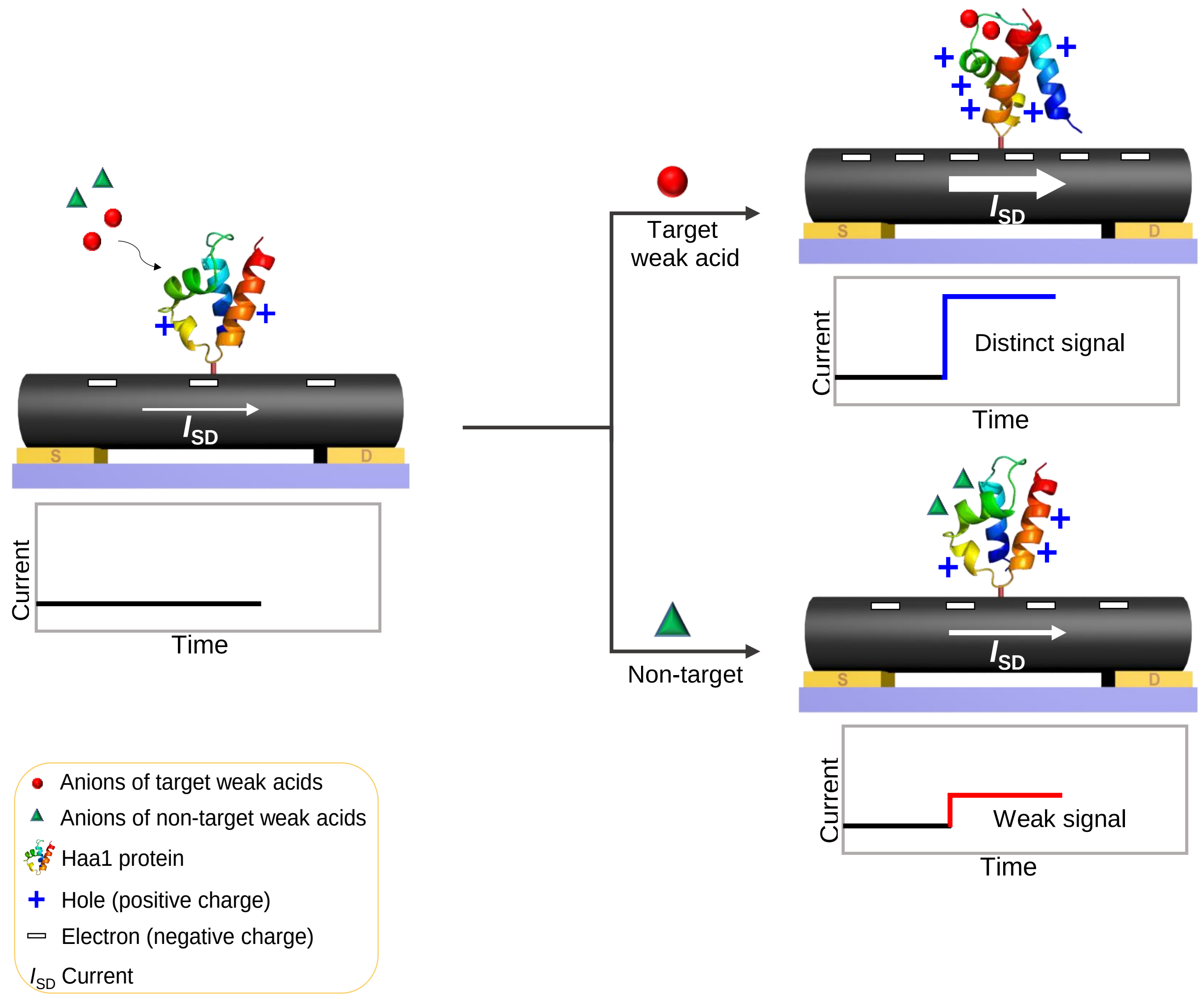


**Figure S11. Schematic illustration of the sensing mechanism of the CNF-FET.** As the immobilized proteins react with weak acids, the charge state around CNF changes and acts as an indirect gate potential. Since CNF-FET exhibits n-type semiconducting behavior, the charge carriers are electrons. When the positive gate voltage is applied to the CNF transducer through solution gate, the gate potential pulls the electrons in the CNF to the surface of the nanofiber, making the effective pathway for electron flow. The interaction between protein (Haa1) and acid anion enhanced positive point charges near the CNF surfaces. This indirect gating effect increases the number of electrons in the CNF and thus increased $I_{SD}$.

## Supplementary references


1. Mumberg, D., Muller, R. and Funk, M. (1995) Yeast vectors for the controlled expression of heterologous proteins in different genetic backgrounds. *Gene*, **156**, 119-122.
2. Uprety, B., Lahudkar, S., Malik, S. and Bhaumik, S.R. (2012) The 19S proteasome subcomplex promotes the targeting of NuA4 HAT to the promoters of ribosomal protein genes to facilitate the recruitment of TFIID for transcriptional initiation in vivo. *Nucleic Acids Research*, **40**, 1969-1983.
3. Cho, B.R., Lee, P. and Hahn, J.S. (2014) CK2-dependent inhibitory phosphorylation is relieved by Ppt1 phosphatase for the ethanol stress-specific activation of Hsf1 in *Saccharomyces cerevisiae*. *Mol Microbiol*, **93**, 306-316.
4. Kim, M.S. and Hahn, J.S. (2016) Role of CK2-dependent phosphorylation of Ifh1 and Crf1 in transcriptional regulation of ribosomal protein genes in *Saccharomyces cerevisiae*. *Biochim Biophys Acta*, **1859**, 1004-1013.
